# Coagulation studies in patients with orthopedic trauma

**DOI:** 10.4103/0974-2700.58652

**Published:** 2010

**Authors:** Kanchana Rangarajan, Arulselvi Subramanian, Jatin S Gandhi, Namit Saraf, Vijay Sharma, Kamran Farooque

**Affiliations:** Department of Laboratory Medicine, Jai Prakash Narayan Apex Trauma Centre, All India Institute of Medical Sciences, New Delhi, India; 1 Department of Orthopedics, Jai Prakash Narayan Apex Trauma Centre, All India Institute of Medical Sciences, New Delhi, India

**Keywords:** Coagulation, DIC scores, fibrinogen, ISS scores, trauma

## Abstract

**Background:**

Head injury, severe acidosis, hypothermia, massive transfusion and hypoxia often complicate traumatic coagulopathy. First line investigations such as prothrombin time, activated partial thromboplastin time, thrombin time, fibrinogen level, platelet count and D-dimer levels help in the initial assessment of coagulopathy in a trauma victim.

**Aim:**

To study the coagulation profile in patients of orthopedic trauma.

**Settings and Design:**

Prospective study.

**Patients and Methods:**

Patients with head injury, severe acidosis, massive transfusion and severe hypoxia were excluded from the study. Coagulation parameters were evaluated at three intervals, at the time of admission, intra operatively and in the postoperative period.

**Statistical Analysis:**

Chi-square test was used for analysis of categorical variables. For comparison between groups, two- way ANOVA was used.

**Results and Conclusions:**

Of the 48 patients studied, 38 (80%) had normal DIC scores upon admission and only 10 (20%) had mild DIC scores at the time of admission. The median Injury Severity Score was 34 and they did not correlate with DIC scores. Fibrinogen levels alone were significantly different, increased progressively (mean pre op, intra op and post op levels 518 ± 31,582 ± 35 and 643 ± 27 respectively; *P* ≤ 0.02) since the time of admission in these patients. All the other parameters remained unchanged. Further large scale prospective studies would be required to correlate elevated fibrinogen levels with the type of trauma or surgery.

## INTRODUCTION

Coagulopathy is one of the major complications of polytrauma and head injury.[1] The factors contributing to post traumatic coagulopathy also include severe acidosis, hypotension, hypothermia, massive transfusion as well as massive tissue injury.[1–4] Severe head injury is one of the most important causes of derangement of coagulation profile post trauma mainly due to the release of tissue thromboplastin. Various studies have convincingly shown that moderate and severe head injury is often complicated by disseminated intravascular coagulation (DIC) and thrombocytopenia.[5–9] Estimation of the coagulation parameters namely prothrombin time (PT), activated partial thromboplastin time (APTT), thrombin time (TT), fibrinogen, D-dimer level as well as platelet counts help in the assessment of impending DIC.[8,10,11] Numerous studies have shown the influence of hypothermia, acidosis and massive transfusion in worsening the coagulation profile and hence the DIC scores.[2–4] However, studies on changes in coagulation profile of orthopedic trauma patients, particularly those with no associated head injury are very few.[12–14] With the present scenario in mind, in this prospective study, we intend to study the coagulation profile in patients of orthopedic trauma.

## PATIENTS AND METHODS

We recruited 48 patients in our study. They were admitted with orthopedic trauma and required active surgical intervention. The study was conducted for a period of six months from July to December 2006.

### Inclusion criteria

1. Stable patients with a GCS score of 14 or higher (no head trauma)

### Exclusion criteria

1. Any prior history of coagulation abnormalities
2. History of intake of drugs like warfarin, epinephrine or systemic disorders like SLE
3. History of liver disease like cirrhosis or any renal impairment
4. History of solid or hematological malignancies
5. Patients with severe hypoxia, hypotension or acidosis at the time of admission

### Lab parameters

On admission, blood was collected by venepuncture in EDTA vacutainers as well as PT tubes containing anticoagulant sodium citrate and processed immediately. This was part of routine protocol followed in a trauma victim in our hospital. We used PT (Neoplastine, Stago, France), APTT (CK prest, stago, France), TT (stago, France), fibrinogen assay (spli prest, stago, France), D- Dimer by semiquantitave Latex agglutination technique (Stago, France). Platelet counts were performed by Beckmann coulter counter- AcT Diff and counterchecked on the slides prepared by Leishmann stain.

Using the criteria outlined by Olson[13] *et al*., the results of all the six hemostatic parameter assessments were graded on a score of zero to three with reference to a range of normal values for a healthy population performed in the same laboratory. The sum of all six assessments for a given patient was regarded as the DIC score. The DIC scores were given grades as normal (zero to three), mild (four to six), moderate (seven to nine), and severe (more than or equal to10).

Coagulation profile was evaluated at three intervals: first, at the time of admission (within first 48 hours), second at the time of surgery and third, at the postoperative period (within 48 hours). Normal, healthy hospital and laboratory staff (normal controls, n=25) with no prior medical illness were matched for age and sex and tested simultaneously with the admission day samples.

### Statistical analysis

Statistical analysis was performed using SPSS 15.0 for Windows (SPSS Inc., Chicago, IL, USA). For analysis of categorical variables, chi square test was used. Comparisons between groups were performed using two ways ANOVA. *P* value < 0.05 was considered significant. For statistical analysis of the D-dimer levels, a consecutive integer from zero to three was assigned to each concentration range. The mean values, rounded to the nearest integer were converted to the appropriate concentration range.

## RESULTS

**Figure 1:**
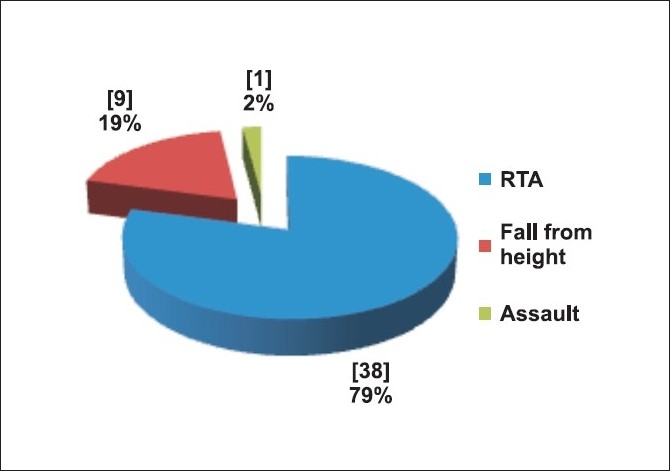
Mode of injury

**Figure 2:**
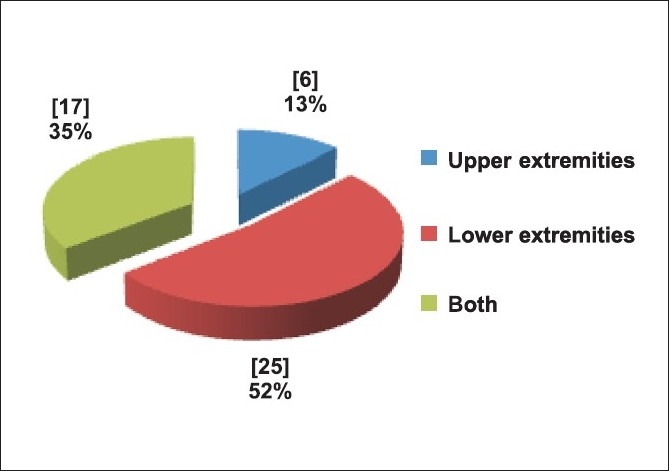
Region of injury

Forty eight patients were included in the study. There were various causes for admission in the hospital [Figure 1]. Patients were of all age ranges (13-80), mean 42 years and predominantly males (89%). The region of injury is shown in [Figure 2].

**Figure 3:**
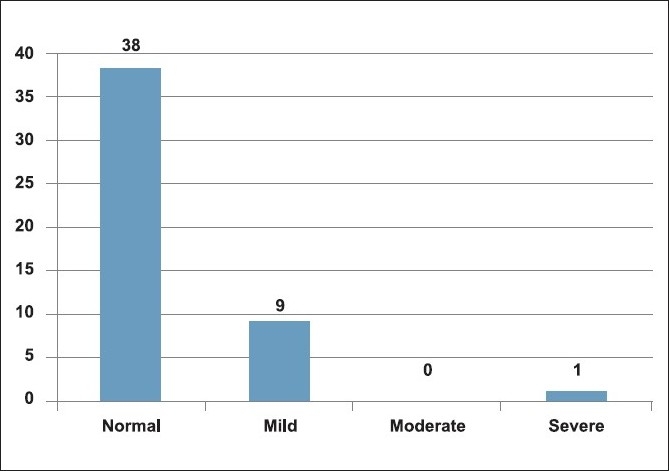
Admission day DIC scores

**Figure 4:**
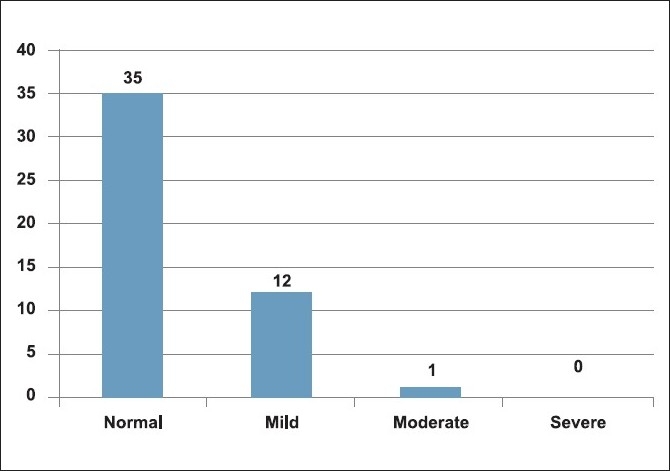
Intra operative DIC scores

The DIC scores at the time of admission and at the time of surgery are shown in Figures 3 and 4. The DIC scores in the postoperative period are also shown [Table 1].

**Table 1:** DIC scores in the postoperative period

N	Normal	Mild	Moderate	Severe
48	34 (70)	12 (26)	02 (4)	0

FIGURES IN PARENTHESIS ARE IN PERCENTAGE

Majority of the patients had normal DIC scores (80%) on the day of trauma and a mild derangement of coagulation profile was present in nine (18%) patients. However, on the day of surgery, mild DIC scores increased to 26%. The DIC scores calculated postoperatively did not show any major change in the coagulation status. Only one patient had severe DIC score and died subsequently due to acidosis and respiratory failure.

The median ISS score was 34 with a minimum score of 24 and a maximum of 54. The mean and standard error of the mean for the various coagulation parameters are shown in Table 2.

**Table 2:** Coagulation parameters at various times of admission

Parameters	Admission day	On the day of surgery	Postoperative period	*P* value<0.05
PT (sec)	14.5±0.38	15.0±0.31	14.40±0.21	0.36
APTT (sec)	30.1±1.0	29.2±0.7	29.8±0.8	0.74
TT (sec)	18.1±0.6	18.0±0.6	18.0±0.4	0.99
Fibrinogen (mg/dl)	518±31	582±35	643±27	0.02
D-dimer (ng/ml)	500-1000	1000-2000	2000-4000	0.18

VALUES GIVEN IN MEAN ± SE

The fibrinogen levels showed significant variation between the three phases. They progressively increased since the time of admission to the postoperative period. The levels of fibrinogen were compared with the injury severity scores and are shown in Table 3. 48.4% of patients with moderate ISS scores (30-44) had high fibrinogen levels while almost half the patients had elevated fibrinogen levels in the preoperative and postoperative period. The *P* value was, however, not significant between these two parameters.

**Table 3:** Fibrinogen levels and ISS scores

ISS	Fibrinogen – pre op		Fibrinogen – per op		Fibrinogen – post op	
			
Grade	N*	H†	N	H	N	H
15-29	6 (35.3)	13 (41.9)	6 (50)	13 (36.1)	1 (25)	18 (40.9)
30-44	10 (58.8)	15 (48.4)	5 (41.7)	20 (55.6)	3 (75)	22 (50)
45-59	1 (5.9)	3 (9.7)	1 (8.3)	3 (8.3)	0 (0)	4 (9.1)

* -NORMAL

† -HIGH, FIGURES IN PARENTHESIS ARE IN PERCENTAGE

The D-dimer levels were also not related to ISS scores. We also compared patient samples with normal controls (n=25) at the time of admission only. All other parameters like PT, APTT, and TT were comparable. We found fibrinogen to be significant (*P*=0.001) in case of patient samples [Table 4]. D-dimer levels in all the normal controls were less than 500 ng/ml.

**Table 4:** Comparison of admission day samples with normal controls

Parameters	Preoperative group (n=48)	Normal controls (n=25)	*P* value<0.05
PT (sec)	14.5±0.38	13.67±0.33	0.156 (NS)
APTT (sec)	30.1±1.0	32.11±0.77	0.115 (NS)
TT (sec)	18.1±0.6	19.51±0.37	0.064 (NS)
Fibrinogen (mg/dl)	518±31	317.26±13.35	0.001 (S)

VALUES GIVEN IN MEAN ± SE; NS - NOT SIGNIFICANT; S - SIGNIFICANT

Most of the variations in platelet count were at the time of injury; Per operative and postoperative changes were very minimal. The platelet counts were analyzed with the ISS scores [Table 5]. The data showed that low platelet counts were associated with increasing ISS scores in four of the patients, remaining normal in majority of them.

**Table 5:** Platelet counts and injury severity scores

Platelet count	ISS scores, *P*<0.01		
	
	15-29	30-44	45-59
Normal	18 (41.9)	23 (53.5)	2 (4.7)
Low	0 (0)	1 (33.3)	2 (66.7)
Very low	1 (50.0)	1 (50.0)	0 (0)

NORMAL-100-400; LOW-50-100; VERY LOW-<50 × 103/μL

## DISCUSSION

In this study, we evaluated patients of isolated orthopedic poly trauma. There was a predominant male population (89.6%). Most of them (79%) were from Delhi and hence were brought to the emergency within a short period of time. Majority of them had road traffic accident (79%) and lower extremity injury (52%). None of the patients had requirement for massive blood transfusion.

The sum of all six parameters namely the DIC score predicts the extent of coagulopathy in patients with multiple traumas and associated head injury.[1] The definitions of coagulopathy have been changed over time, and in addition, other techniques for measuring DIC have become available in the last decade.[15] DIC scores have been used to assess the coagulation status of a patient in many studies.[13–15] The lab diagnosis of DIC was recently simplified by the International Society of Thrombosis and Hemostasis (ISTH).[15] The score is based on platelet count, elevated fibrin-related marker, prolonged PT and fibrinogen level.

In the present study, DIC scores were only minimally deranged (18%) at the time of admission and increased slightly with surgical intervention (26%) and remained the same postoperatively (26%). There was however one patient who had severe derangement of coagulation profile (severe DIC score), a poor outcome due to acidosis and respiratory failure. All the other patients had a good outcome and were discharged from the hospital.

According to Brohi *et al*.,[16] clinically important coagulopathy developed upon admission in patients with ISS greater than 15. The authors assessed the possibility of coagulopathy in patients with multiple injuries which also included head injury. In this retrospective analysis of 1079 patients, only 23.7% had evidence of coagulopathy upon admission. Our study examines the possibility of coagulopathy in trauma patients in the absence of preexisting medical conditions. We presume that severe coagulation changes are not a finding in our population considering a cut off of PT more than 20.5 sec, APTT more than 48 sec and TT more than 29 sec. This may be one of the reasons why our patients with high median ISS scores more than 34 did not present with severe coagulopathy upon admission.

Seyfer *et al*.[17] compared three groups of patients in their study. The first group of patients had no preexisting medical illness and had normal coagulation profile. They underwent routine elective orthopedic procedures. This set of patients had fall in the antithrombin -3, plasminogen as well as APTT and all the values including PT, APTT and TT reverted to normal within 24 hours postoperatively. We believe our study group is similar and hence no major changes in coagulation profile were seen intra and postoperatively.

On comparing the ISS scores with the fibrinogen levels, it was seen that majority of patients in the preoperative (90.3%), per operative (91.7%) and postoperative (90.9%) period had high fibrinogen levels correlating with increasing ISS scores (15-44). These points to the fact that increased severity of injury as seen by high ISS scores correlated with high fibrinogen levels. Other parameters like PT, APTT, TT and D-dimer levels did not correlate with ISS scores.

About 89.5% had normal platelet counts irrespective of high ISS scores. Studies have shown that thrombocytopenia is a usual consequence of coagulopathy associated with severe trauma (ISS more than 25).[16,18] Thrombocytopenia was seen in only three of our patients with high ISS scores. Only one out of forty eight patients had very low platelet counts (less than 50,000 ×10^3^/cu mm) corresponding to ISS scores more than 25.

We also compared patient samples with the control samples at the time of admission only. All the parameters like PT, APTT and TT were comparable. However, fibrinogen levels were altered post trauma and continued to increase over a period of time.[19,20,21] There was also a significant change in the fibrinogen levels (*P*=0.02) since the time of admission and increased progressively on the day of surgery and raised also in the postoperative period. This showed that fibrinogen was the only parameter which was affected post trauma.

### Strengths of the study

This study is one of the first of its kinds analyzing coagulation profile changes in isolated orthopedic trauma patients. Though our sample size was small, one important finding was correlation of high injury severity scores with high fibrinogen levels. The progressive increase in the fibrinogen levels since the time of admission was another finding which cannot be easily overlooked.

### Limitations

The admission day samples were compared with healthy, normal controls. The coagulation parameters like PT, APTT and TT were not significant between control samples and admission day samples. However, only fibrinogen was significantly affected in the admission day samples (*P*=0.001).This may be due to the small sample size of our study population. Further, controls were compared only with the admission day samples; they could not be compared during and after surgery.

### Future directions

Large scale prospective trials are underway to see the coagulation profile changes in patients with both orthopedic trauma as well as head injury. Future large scale trials are being done to see the consequences of high fibrinogen levels and effect of such raised levels over a period of time. Certain effects of increased fibrinogen like deep vein thrombosis in these trauma victims are also being studied.

## CONCLUSIONS

Of the 48 patients studied, we had 38 patients (80%) with normal DIC scores upon admission and only 10 patients (20%) with mild DIC scores at the time of admission. The median Injury Severity Score was 34 and they did not correlate with DIC scores. Fibrinogen levels alone were significantly raised, increased progressively (mean pre op, intra op and post op levels 518 ± 31,582 ± 35 and 643 ± 27 respectively; *P* ≤ 0.02) since the time of admission in these patients. All the other parameters remained unchanged. Also, increased severity of injury as seen by high ISS scores correlated with high fibrinogen levels. Further large scale prospective studies would be required to correlate elevated fibrinogen levels with the type of trauma or surgery and the effect of such high fibrinogen levels on these trauma patients.
